# Application of untargeted volatile profiling in inflammatory bowel disease research

**DOI:** 10.1007/s00216-023-04748-x

**Published:** 2023-05-26

**Authors:** Natalia Arroyo-Manzanares, María García-Nicolás, Fuensanta Abellán-Alfocea, Laura Prieto-Baeza, Natalia Campillo, Blanca del Val Oliver, José Zarauz-García, Luis Sáenz, Pilar Viñas

**Affiliations:** 1 Department of Analytical Chemistry, Faculty of Chemistry, University of Murcia, Regional Campus of International Excellence “Campus Mare Nostrum”, E-30100 Murcia, Spain; 2 Internal Medicine Service - Gastroenterology and Hepatology Section, Hospital General Universitario Rafael Méndez, Lorca, Spain; 3 Laboratory Medicine Department, Hospital General Universitario Rafael Méndez, Lorca, Spain

**Keywords:** Inflammatory bowel disease, Headspace, Gas chromatography–mass spectrometry, Untargeted metabolomic strategy, Chemometrics

## Abstract

Inflammatory bowel disease (IBD) diagnosis depends on criteria based on histological, endoscopic, radiological, and clinical results. These studies show drawbacks as being expensive, invasive, and time-consuming. In this work, an untargeted metabolomic strategy based on the monitoring of volatile compounds in serum by headspace gas chromatography–mass spectrometry is proposed as a complementary, fast, and efficient test for IBD patient diagnosis. To develop the method and build a chemometric model that allows the IBD diagnosis, serum samples including IBD patients and healthy volunteers were collected. Analyses were performed by incubating 400 µL of serum for 10 min at 90 °C. For data processing, an untargeted metabolomic strategy was used. A total of 96 features were detected, of which a total of 10 volatile compounds could be identified and confirmed by means of the analysis of real standards. The chemometric treatment consisted of a discriminant analysis of orthogonal partial least squares (OPLS-DA) obtaining a 100% of classification rate, since all the analyzed samples were correctly classified.

**Graphical abstract:**

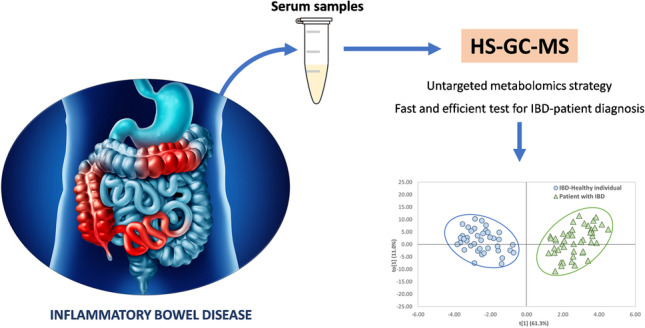

**Supplementary information:**

The online version contains supplementary material available at 10.1007/s00216-023-04748-x.

## Introduction

Inflammatory bowel disease (IBD) is a chronic, multifactorial immune disorder characterized mainly, but not exclusively, by inflammation of the intestine. In general, IBD encompasses two different pathologies: ulcerative colitis (UC) and Crohn’s disease (CD). Clinically, similar symptoms are shared for both diseases, such as bloody diarrhea, abdominal pain, weakness, and weight loss, while they tend to differ in the complications and prevalence, as well as the location and depth of inflammation [1, 2]. In cases where it is impossible to differentiate between UC and CD, since patients show overlapping pathological features of UC and CD, it often results in an interim diagnosis of unclassified IBD or indeterminate colitis (IC). There is a fourth entity, named microscopic colitis (MC), often misdiagnosed and confused with irritable bowel syndrome by not producing obvious macroscopic lesions.

There is no specific test for IBD diagnosis. In addition, the disease occurs in a fluctuating way, with periods of inflammatory flare-ups alternating with other periods of inflammatory remission. First, clinical symptoms are examined, including anemia, abdominal pain, weakness, weight loss, and diarrhea with blood and/or mucus. In general, the clinical suspicion of IBD prompts the practice of blood and stool tests, colonoscopy, and some imaging studies that confirm the diagnosis excluding other causes.

Analysis of blood can show an elevated white blood cell count and elevated C-reactive protein (CRP) levels. CRP is a plasma protein synthesized in the liver that can be used as a marker for indirect determination of the degree of inflammation in IBD. Under normal circumstances, CRP is synthesized in small amounts (< 1 mg L^−1^); however, because of some stimuli, usually inflammation, hepatocytes rapidly increase their synthesis [2, 3].

Regarding stool analysis, calprotectin is an abundant and widely distributed protein, mainly found in monocytes, reactive macrophages, and polymorphonuclear leukocytes. Its biological function is not exactly known, but its protective activity in inflammatory, proliferative, and infectious processes stands out, in which its plasma levels are increased from 8 to 40 times. Therefore, calprotectin determination in feces has been recently proposed as a new marker for the diagnosis of IBD [4, 5], which can be used to predict the risk of relapse or monitor response to treatment. In addition, it offers greater sensitivity and specificity than CRP.

An elevated level of calprotectin (> 50 µg g^−1^) identifies patients who are more likely to have IBD and who should undergo colonoscopy. In contrast, a fecal calprotectin level  < 50 µg g^−1^ makes the diagnosis of IBD highly unlikely. Colonoscopy allows direct visualization of the intestinal mucosa and obtaining biopsies, making it the preferred test for IBD diagnosis. However, it has some risks and limitations, since it is an invasive procedure with relatively high cost and requires bowel preparation and patient sedation. Furthermore, it is estimated that more than 60% of colonoscopies performed in young patients are potentially preventable since their results are normal [6].

In addition, disease status often needs to be reassessed with complementary explorations that can safely, quickly, and reliably detect the presence of inflammation. In the case of radiological tests, in patients with CD, magnetic resonance imaging with bowel contrast is preferable to computerized tomography scan since its diagnostic precision is greater and avoids frequent exposure to radiation.

Thus, the diagnosis of IBD depends on criteria based on the results of clinical, endoscopic, histological, and radiological examinations, which are often costly, invasive, and time-consuming. Therefore, low-cost, rapid, and non-invasive diagnostic tools are needed. In recent years, metabolomics approaches have been showing promising results in the diagnosis of IBD. The metabolomic analysis consists of the exhaustive and quantitative study of the metabolome, understanding this as the complete set of small molecules called metabolites that are synthesized by a biological system [7]. Sample selection in metabolomics studies is key, as each type of sample provides different biochemical information: blood can provide useful information on systemic metabolism, while fecal profiles are indicative of digestive metabolism, and urine provides a profile of endogenous metabolism. Due to the chemical diversity of metabolites, the choice of analytical technique will also be decisive in the metabolomic study.

To date, proton nuclear magnetic resonance spectroscopy (^1^H-NMR) is the most widely used technique in metabolomics studies for IBD diagnosis. Studies have been conducted on biofluids such as plasma, serum, feces, and urine from patients with this disease and have been compared with IBD-healthy individuals. These studies have focused primarily on non-complex and small molecules such as amino acids or derivative metabolites. It has been reported that there are differences in metabolic profiles between IBD patients and healthy controls [8–13], as well as between IBD subtypes [8, 9, 13].

Liquid chromatography with tandem mass spectrometry (LC-MS/MS) has also been applied for monitoring the inflammation lipid mediator leukotriene E4 in plasma [14], and the lipoid profile in urine [15] as biomarkers of IBD activity. Fourier transform ion cyclotron resonance mass spectrometry (FT-ICR-MS) has also been applied for the analysis of fecal samples in patients with CD [16]. Recently, the potential of LC coupled to high-resolution mass spectrometry (HRMS) has also been investigated, demonstrating that there is a loss of “metabolic diversity” among IBD patients since gut metabolites were frequently depleted, thus leading to alterations in immune response and cell proliferation, among other biological processes [17].

Gas chromatography coupled to mass spectrometry (GC-MS) has allowed the analysis of volatile metabolites in serum [18] or feces [19] as an assessment approach to IBD or its subtypes. Specifically, Kohashi et al. [18] mainly targeted water-soluble metabolites for UC diagnostic, reporting that the components of the urea cycle (citrulline, ornithine, and urea) and the tricarboxylic acid cycle (fumaric acid, succinic acid, and malic acid) showed the most significant alterations, and all of them except urea exhibited a significant decrease of serum levels in the UC patients. In addition, significant decreases in serum levels of several amino acids such as isoleucine, lysine, histidine, leucine, and methionine have been detected in UC patients. Ahmed et al. [19] monitored between 234 and 290 metabolites to discriminate inactive and active CD or UC. The statistically significant metabolites in separating the groups were broadly classified into aldehydes, ketones, secondary alcohols, esters, and short and branched-chain fatty acids. More recent studies focus on the analysis of fecal [20] and exhaled volatile organic compounds (VOCs) [21] using GC coupled to ion mobility spectrometry (IMS).

The goal of this work is to investigate a totally untargeted metabolomic approach based on the total VOC profile obtained by GC-MS for the diagnosis of IBD. The novelty includes that a headspace (HS) analysis is proposed as a sample treatment of serum and introduction system, avoiding more complex procedures proposed to date, which are based on solid-phase microextraction (SPME) [18] or the combination of liquid-liquid extraction and lyophilization and derivatization steps [17]. On the other hand, this is the first study carried out in a totally untargeted mode.

## Material and methods

### Instrumentation and software

HS-GC-MS analyses were carried out on an 8890 gas chromatograph (Agilent Technologies, Santa Clara, CA, USA) equipped with an HS system (Gerstel, Mülheim, Germany) and coupled to a mass spectrometer (5977B) quadrupole mass selective detector (Agilent Technologies) with inert ion source based on electron impact (EI). The chromatographic separation was carried out on an HP-5MS capillary column (30 m × 0.25 mm inner diameter × 0.25 µm film thickness), also from Agilent Technologies.

Analysis and data acquisition were carried out using the MSD Chemstation Data Analysis application (Version G1701EA), and data were processed using MS-DIAL (Version 4.80, RIKEN) and SIMCA-P (Umetrics, Malmö, Sweden).

### Standards and reagents

A total of 10 analytical standards were used for the identification of volatile compounds in serum samples: butanal, pentanal, toluene, hexanal, ethylbenzene, p-xylene, heptanal, 1-ethyl-3-methylbenzene, 1,3,5-trimethylbenzene, and 2-ethyl-1-hexanol. All of them were supplied by Sigma-Aldrich (St. Louis, MO, USA) and individual stock solutions were prepared at 1000 µg mL^−1^ in methanol (MeOH). All standard solutions were stored at  − 20 °C.

High-quality MeOH was provided by ChemLab (Zedelgem, Belgium). The carrier gas used was helium with 99.99% purity and was provided by Air Liquide (Madrid, Spain).

A mixture of alkanes (from C8 to C40) at 500 µg mL^−1^ in dichloromethane was also supplied by Sigma-Aldrich. The alkane mixture was prepared at 1 µg mL^−1^ in hexane and used as quality control throughout the analyses.

### HS-GC–MS method

A volume of 400 µL of sample was incubated at 90 °C for 10 min, while being stirred at 750 rpm. Then, 1.5 mL of the headspace was injected into the GC system using a 2.5 mL syringe (90 °C) in splitless mode. The carrier gas was helium with a flow rate of 1 mL min^−1^. The program oven was as follows: initial temperature of 40 °C held for 5 min, which was increased to 130 °C at 5 °C min^−1^ and subsequently to 200 °C at 35 °C min^−1^ (total run 25 min). The MS was operated in EI mode at 70 eV. Temperatures of the ion source, transfer line, and quadrupole were set at 230, 280, and 150 °C, respectively. Data acquisition was carried out in the range of 20–400 m*/z.*

### Samples

The ethics committees of Murcia University (Favourable Report ID: 2908/2020) and Rafael Méndez University Hospital (Lorca, Spain) approved this study. Informed consent was obtained from all patients and volunteers, and samples were used in accordance with the hospital guidelines.

A total of 56 patients with IBD and 48 volunteers with no suspicion of IBD disease were recruited from the Rafael Méndez University Hospital. IBD diagnosis was established by endoscopic, histological, and radiological criteria. Since IBD is a very broad disease, the patients who participated in this study were selected with different clinical pictures to cover a wide spectrum of the disease.

Table 1 shows the clinical and demographic characteristics of the study population. The study cohort comprised 55% females in the IBD-healthy volunteer’s group and 64% females in patients with IBD. The mean age of cohort patients with IBD disease and IBD-healthy volunteer was 48.9 and 43.0, respectively. Table 1 also shows CRP, calprotectin, and other parameters such as cholesterol, Fe, and hemoglobin (HGB), since they had been previously described as abnormalities exhibited by patients with IBD [22, 23], although no significant differences were found between the two groups. It should be noted that some IBD-healthy volunteers showed high CRP, suggesting another inflammatory process, not related to IBD.

**Table 1:** Clinical and demographic characteristics of the study population

	IBD-healthy volunteers	Patients with IBD	*P*-value*
Number of participants	45	56	
Gender			
Male, *n* (%)	20 (45)	20 (36)	
Female, *n* (%)	25 (55)	36 (64)	
Age (years)			
Median (IQR)	43.0 (23.0–63.4)	48.9 (38.2–57.1)	0.250
Cholesterol (mg/dL)			
Median (IQR)	186.0 (161.5–208.0)	192.5 (170.5–215.0)	0.810
Missing (%)	11	5	
Fe (μg/dL)			
Median (IQR)	86.3 (83.0–100.5)	84.8 (56.0–100.0)	0.740
Missing (%)	51	64	
HGB (g/dL)			
Median (IQR)	13.6 (13.0–14.5)	14.1 (13.3–15.1)	0.584
Missing (%)	7	14	
LEU (mg/dL)			
Median (IQR)	6.2 (6.1–7.6)	7.5 (5.7–8.6)	0.571
Missing (%)	9	18	
CRP (mg/dL)			
Median (IQR)	0.5 (0.1–0.8)	0.7 (0.1–0.6)	0.280
Missing (%)	16	2	
CAL (µg/g)			
Median (IQR)	88.6 (68.0–127.0)	297.4 (29.5–287.4)	0.350
Missing (%)	10	3	

*HGB* hemoglobin, *LEU* leucocytes, *CRP* C-reactive protein, *CAL* calprotectin, *IQR* interquartile range

*Both groups were compared using a *t* test

IBD patients who participated in the study received a wide variety of treatments, including those specific to the inflammatory disease such as aminosalicylates (mesalazine), thiopurines (azathioprine), corticosteroids (budesonide, beclomethasone dipropionate, prednisone, and methylprednisolone), and biological (anti-TNF, anti-interleukins IL12/23, anti-integrins, JAK inhibitors…) as well as mineral (iron or calcium) and vitamin (folic acid, cyanocobalamin, calciferol) supplements. In the case of the IBD-healthy volunteers, some of them also had some type of medication including antihypertensives, antibiotics, analgesics, antipsychotics, or antidepressants.

Each blood sample was collected in a special tube for blood collection. This tube contains a separating gel that allows the cells to be separated from the blood, obtaining serum as the final result after being centrifuged at 3000 rpm for 10 min at room temperature. Then, the serum was transferred to a clean tube and stored at  − 20 °C until use.

For their analysis, serum samples were tempered at room temperature for approximately half an hour and vigorously vortexed for 2 min for sample homogenization. Then, 400 µL of serum was placed into a 20 mL glass vial and hermetically sealed with 18 mm aluminum screw caps provided with silicone septum and injected in the HS-GC-MS system.

### Data processing

Data from HS-GC-MS were converted to analysis Base Framework (ABF) formats and processed using MS-DIAL, which includes peak picking, deconvolution, compound identification, and peak alignment. The peak identification was carried out using an alkane mix–based retention index by Kovats’s method and GC-MS metabolomics MSP spectral library (RIKEN).

The features (including both identified and unidentified compounds) were used to carry out the multivariate study. The chemometric analysis consisted of a discriminant analysis of orthogonal partial least squares (OPLS-DA) using a unit variance (UV) scale and was carried out using SIMCA-P software. The OPLS-DA model was built using 80% of the samples and was externally validated with the remaining 20%. The ellipses for each category using a confidence probability level of 95% were obtained with Excel software using available online algorithms. R2X(cum), R2Y(cum), Q2(cum), and classification rate (CR) were evaluated in order to study the model success. The cross-validation procedure was a sevenfold cross-validation without shuffling. R2X(cum) and R2Y(cum) represent the cumulative fraction of the variance explained by a specific component. Q2(cum) indicates the predictive ability of the chemometric model, which should have a value greater than 0.5 [24]. In addition, validation of the model was carried out using a permutation test using 50 random permutations.

Quality control samples were included at the start and end of an assay run, and at regular intervals throughout the assay (one QC every 5 samples analyzed). QC was a mixture of serum samples with the aim of obtaining a representative sample of the qualitative and quantitative composition of the samples. Principal component analysis (PCA) was carried out to check possible signal deviations or sensitivity loss.

## Results and discussion

### Optimization of HS-GC–MS method

The optimization of the HS-GC-MS method was carried out with the aim of achieving the best results in terms of intensity and separation between peaks. Specifically, oven program, volume of serum sample, and temperature and time of incubation were investigated.

Considering that the objective of an untargeted metabolomic method is to obtain as much information as possible, it is essential to achieve a good separation between peaks in the shortest possible time, so the first parameter optimized was the oven program. Among the different temperature programs assayed, the best results were obtained with the following conditions: initial temperature of 40 °C held for 5 min, which was increased to 130 °C at 5 °C min^−1^ and subsequently to 200 °C at 35 °C min^−1^, thus providing a total run time of 25 min.

**Fig. 1:**
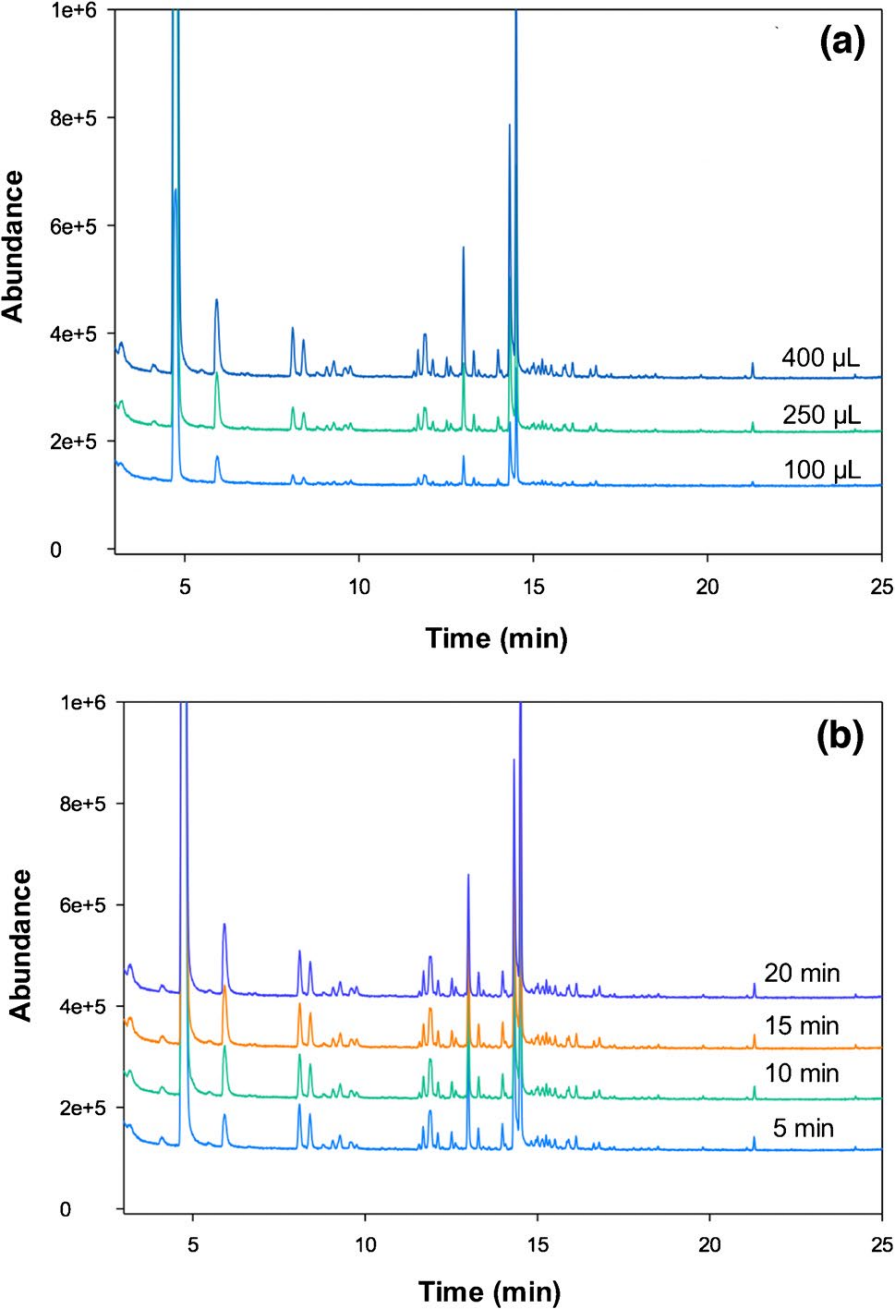
Optimization of **a** volume of sample, **b** time of incubation

The amount of the sample available limited the range of sample volumes investigated. Experiments were carried out using 100, 250, and 400 µL of serum. As expected, using the greater amount of sample, the number of signals and their intensity increased (Fig. 1a). Thus, 400 µL was selected for further experiments.

Next, the effect of the incubation temperature was investigated. Experiments at temperatures between 80 and 100 °C were performed. When the temperature increased from 80 to 90 °C, the intensity of the signals also increased. This is because high temperatures facilitate the release of volatile organic compounds with high boiling points. However, when the temperature increased from 90 to 100 °C, practically no significant differences were observed, for which reason 90 °C was finally selected as the optimum value.

Finally, the incubation time was studied between 5 to 20 min. As can be seen in Fig. 1b, the time range studied did not have a very significant effect on the intensity of the signals obtained. A small increase was observed in the signals that appear at the beginning of the chromatogram when increasing the time from 5 to 10 min. However, after 10 min, no differences were observed. An incubation time of 10 min was therefore selected as optimum.

### Metabolic features derived from untargeted metabolomic analysis and peak identification

For data analysis, an untargeted metabolomic strategy was proposed using MS-DIAL. According to the acquisition method, the mass range was established between 20 and 400 m*/z*. For peak detection, data points were smoothed with a linearly weighted smoothing average using a level of 3 scans and an average peak width of 20 scans. Noise was defined by ion amplitude less than 1000. The deconvolution was carried out to the detected *m/z*–retention time features, thus features with identical peak widths and retention times were combined into single arrays. In this case, a resolution of 0.5 was set, since higher values could decrease the number of resolved chromatographic peaks and lower values could recognize noise as chromatographic peaks.

For peak identification, an alkane mix–based retention index by Kovats’s method was used. The alkane mix (C_8_ to C_40_) was analyzed using the proposed methodology and a dictionary with retention time was created and used for identification purposes. Deconvoluted spectra were matched against a GC-MS metabolomic MSP spectral library from RIKEN. The retention index, retention time, and *m/z* tolerances were set at 20, 0.5 min, and 0.5 Da, respectively. A match criterion of more than 85% was considered. Finally, alignment was carried out using a retention time tolerance of 0.075 min and spectral similarity tolerance of 70%.

**Fig. 2:**
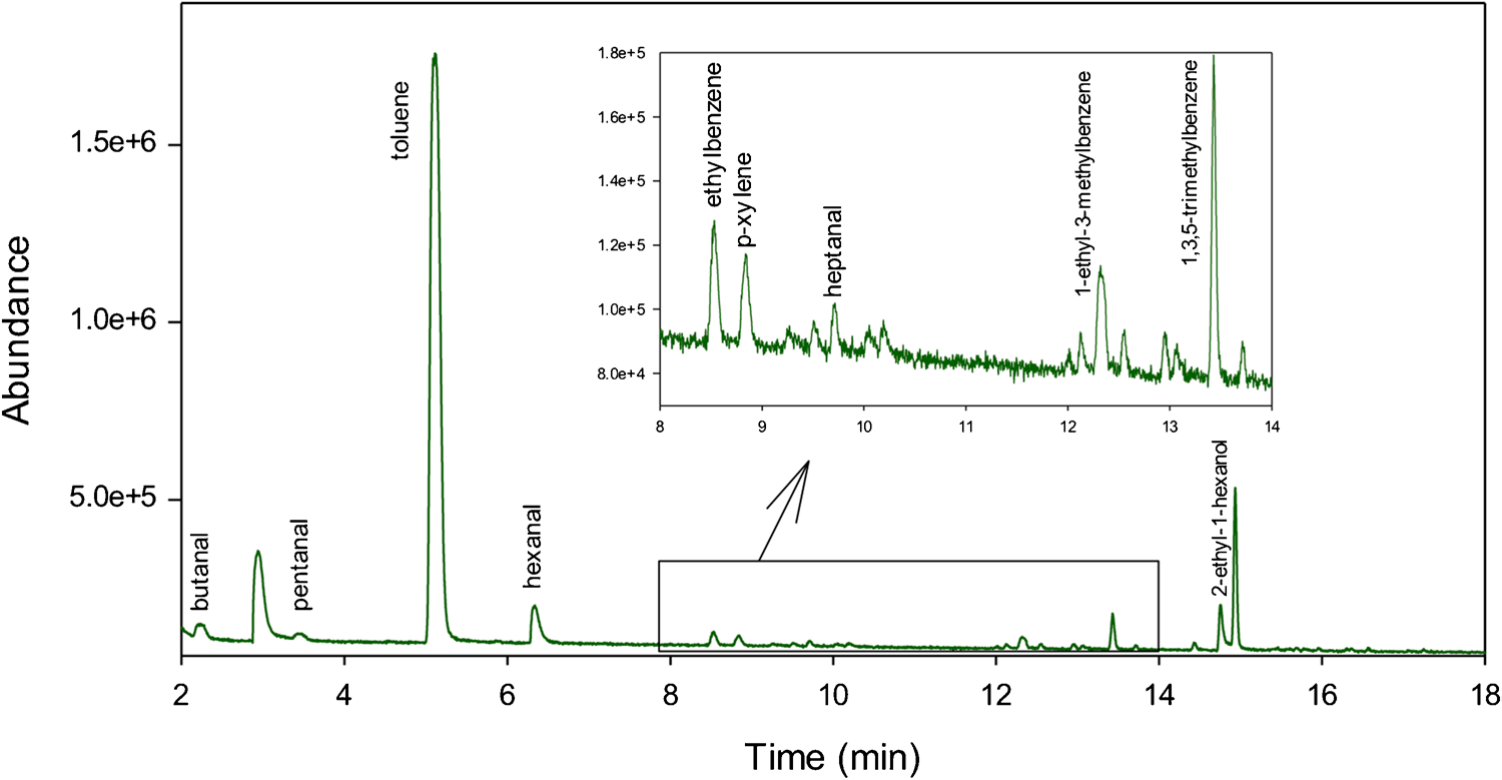
HS-GC-MS chromatogram of a serum human sample showing the identified compounds

A total of 96 features were detected, of which a total of 10 compounds could be identified and confirmed by means of the analysis of real standards (Fig. 2). Table 2 summarizes the identified compounds.

**Table 2:** Detected compounds in serum samples

RT (min)	Compound	Ions (*m/z*)
2.3	Butanal	44, 43, 72, 41
3.5	Pentanal	44, 58, 29, 41
5.1	Toluene	91, 92, 77, 65
6.3	Hexanal	56, 44, 41, 43
8.5	Ethylbenzene	106, 91, 65, 77
8.8	p-Xylene	91, 106, 105, 77
9.8	Heptanal	70, 41, 44, 43
12.3	1-Ethyl-3-methylbenzene	120, 105, 91, 77
13.4	1,3,5-Trimethylbenzene	105, 120, 91, 77
14.8	2-Ethyl-1-hexanol	57, 41, 43, 70, 83

The presence of butanal, pentanal, toluene, hexanal, ethylbenzene, p-xylene, and heptanal has been previously described in serum human samples [25, 26]. 1-Ethyl-3-methylbenzene has been associated with smokers and has been detected in exhaled breath [27], while 1,3,5-trimethylbenzene and 2-ethyl-1-hexanol have been detected in biological human and rat samples after exposure to these compounds [28, 29].

Subsequently, a statistical study of the identified compounds in the samples was carried out. Therefore, a *t* test was performed using the peak area values of the samples of each category since data fit a normal distribution. Only pentanal (*p*-value = 0.01) and hexanal (*p*-value = 0.01) presented statistically significant differences between the mean from IBD-healthy volunteers and IBD patients at the 95.0% confidence level. However, no significant differences were obtained for butanal, toluene, p-xylene, heptanal, 1-ethyl-3-methylbenzene, 1,3,5-trimethylbenzene, and 2-ethyl-1-hexanol (*p*-value  > 0.05).

### Chemometric models for the identification of IBD patients

The 96 features (including known and unknown compounds) were used to create the dataset for multivariate analysis, so it had a dimension of 101 (samples) × 96 (features). The 101 samples included both serum samples of IBD-healthy volunteers (45 samples) and serum samples of patients with IBD (56 samples).

Initially, the residual normal probability plot was obtained. The residuals were random and normally distributed, since the normal probability plot has all the points lying on a straight line. The normal distribution of the data makes it possible to create the models using the raw data without transformations. In addition, all experimental runs ranged between  − 2.5 and  + 2.5; therefore, no outliers were detected (Supplemental Figure S1).

PCA was firstly applied over the training set to visualize any possible grouping of samples, but it was not enough to discriminate the categories (Supplemental Figure S2). Therefore, an OPLS-DA using UV scale or autoscaling, the most applied in metabolomics and that uses the standard deviation as the scaling factor, was proposed [30].

**Fig. 3:**
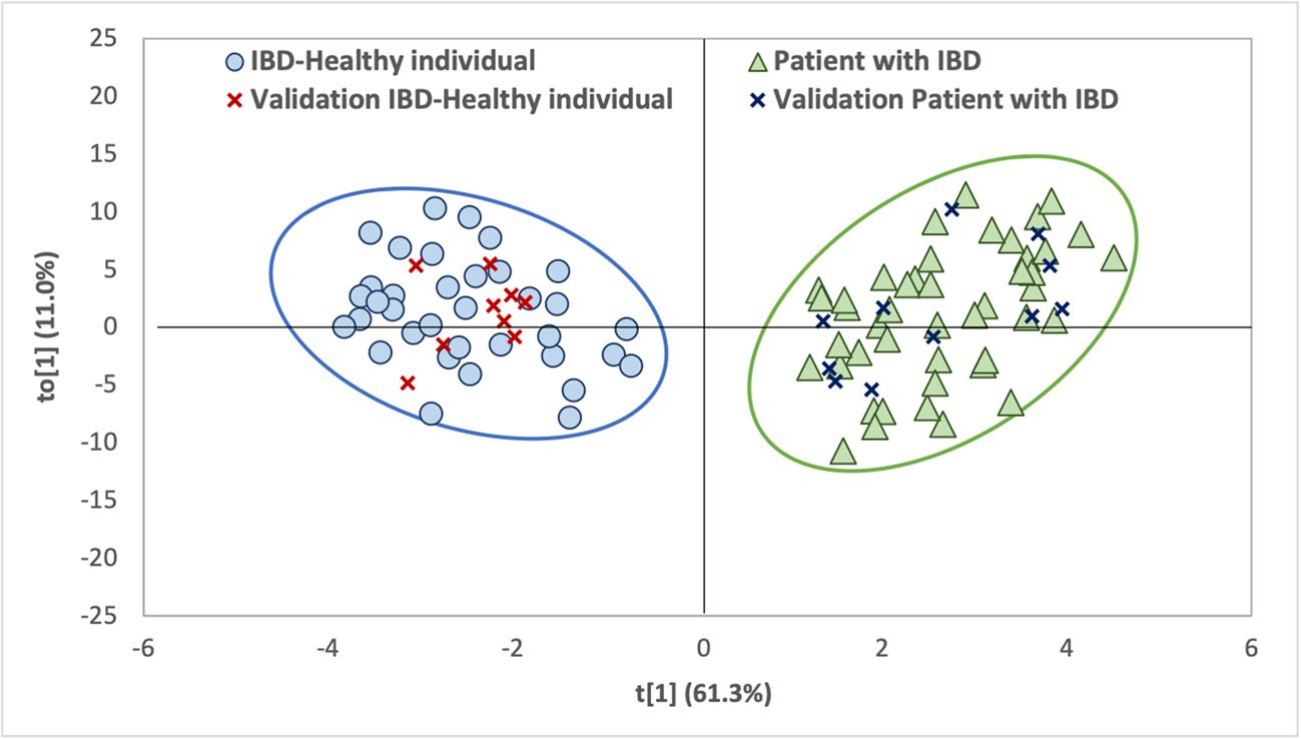
OPLS-DA score plot for the classification of serum samples of IBD-healthy and IBD patients including validation set (predicted samples). Ellipses for each category using a confidence probability level of 95% are shown

For the construction and validation of the models, the dataset was randomly divided into two subsets. Eighty percent of samples were used for the model optimization (45 serum samples from patients with IBD and 36 from IBD-healthy volunteers) and the remaining 20% for its validation (11 serum samples from patients with IBD and 9 from IBD-healthy volunteers). The obtained chemometric model (1 + 1 component, R2X = 0.828, R2Y = 0.723, and Q2 = 0.543) perfectly separated the samples from patients with IBD and IBD-healthy volunteers as can be seen in Fig. 3. Q2 > 0.5 is usually admitted for good predictability, although it is difficult to give a general limit that corresponds to good predictability, since this strongly depends on the properties of the dataset. For this reason, in order to demonstrate the model predictability, CV-ANOVA was carried out (Supplemental Table S1) and the model has also been validated by a permutation test that consists of comparing the Q2 obtained for the original dataset with the distribution of Q2 values calculated when original Y values are randomly assigned to the individuals [31]. Supplemental Figure S3 shows the permutation plot for the OPLS-DA model using 50 random permutations. This plot confirms the validity of the model since all Q2 and R2 values to the left are lower than the original points to the right and the regression line of the Q2-points intersects the vertical axis below zero. Moreover, after applying the model to the validation set, a CR of 100% was obtained, since all samples were correctly classified. The OPLS-DA models met all quality criteria; it was proposed due to its ability to model data with noisy and multicollinear variables, such as spectral metabolic data. However, its results were also compared with those obtained using a PLS-DA model (Supplemental Information), demonstrating that the OPLS-DA allows obtaining a better separation between the samples of patients with IBD and IBD-healthy volunteers.

The variable influence on the projections (VIP) plot (Supplemental Figure S4) revealed that hexanal and pentanal were two of the compounds that most influenced the classification of the samples, in agreement with the data obtained from the ANOVA analysis. Further modeling of men and women separately allowed us to evaluate the influence of the confounding factors by means of shared and unique structures (SUS) plot. The SUS plot compared the contribution of volatile compounds to differentiate IBD-healthy females and females with IBD versus IBD-healthy males and males with IBD (Supplemental Figure S5). The majority of features are closely clustered around the diagonal of the SUS plot; therefore the volatile compounds responsible for the differentiation of patients with IB are the same in women and men, which is an added value to the proposed methodology.

## Conclusions

This work proposes an analytical strategy based on the use of HS-GC-MS and its combination with chemometric models as a possible complementary tool for the diagnosis of patients with IBD.

The untargeted metabolomic strategy allowed us to obtain 96 features that were used to build an OPLS-DA model that would allow us to separate patients with IBD and IBD-healthy individuals. The external validation did not give rise to false positives or negatives (100% of the classification rate). The results showed a higher concentration of hexanal and pentanal in patients with IBD. In addition, a similar volatile profile was found for female and male IBD patients.

The main advantages of the proposed method are its speed in obtaining reliable results, since it takes less than an hour to apply the HS-GC-MS method and the chemometric model, and the no need for sample treatment, since the volatiles generated in the headspace when heating the sample are monitored.

Although the results obtained demonstrate the potential of this methodology, it should continue to be investigated increasing the number of samples, and in order to demonstrate its applicability for the differentiation of patients with CD or UC and evaluate the influence in the classification of the other inflammatory diseases.

## Supplementary Information

Below is the link to the electronic supplementary material.

Supplementary file1 (DOCX 421 KB)

## Data Availability

Not applicable.
