# Supplementary material for: Application of untargeted volatile profiling in inflammatory bowel disease research
Source: Anal Bioanal Chem. 2023 May 26;415(17):3571–9. doi: 10.1007/s00216-023-04748-x (PMC10289974; doi:10.1007/s00216-023-04748-x)
Supplement: Supplementary file 1 — Supplementary file1 (DOCX 421 KB) [file 216_2023_4748_MOESM1_ESM.docx]

**Application of untargeted volatile profiling in inflammatory bowel disease research**

Natalia Arroyo-Manzanares^1^ • María García-Nicolás^1^ • Fuensanta Abellán-Alfocea^1^ • Laura Prieto-Baeza^1^ • Natalia Campillo^1^ • Blanca del Val Oliver^2^ • José Zarauz-García^3^ • Luis Sáenz^3^ • Pilar Viñas^1^

^1^Department of Analytical Chemistry, Faculty of Chemistry, University of Murcia, Regional Campus of International Excellence "Campus Mare Nostrum", E-30071, Murcia, Spain.

^2^Internal Medicine Service - Gastroenterology and Hepatology Section, Hospital General Universitario Rafael Méndez, Lorca, Spain.

^3^Laboratory Medicine Department, Hospital General Universitario Rafael Méndez, Lorca, Spain.

* Corresponding author: [pilarvi@um.es](mailto:pilarvi@um.es)


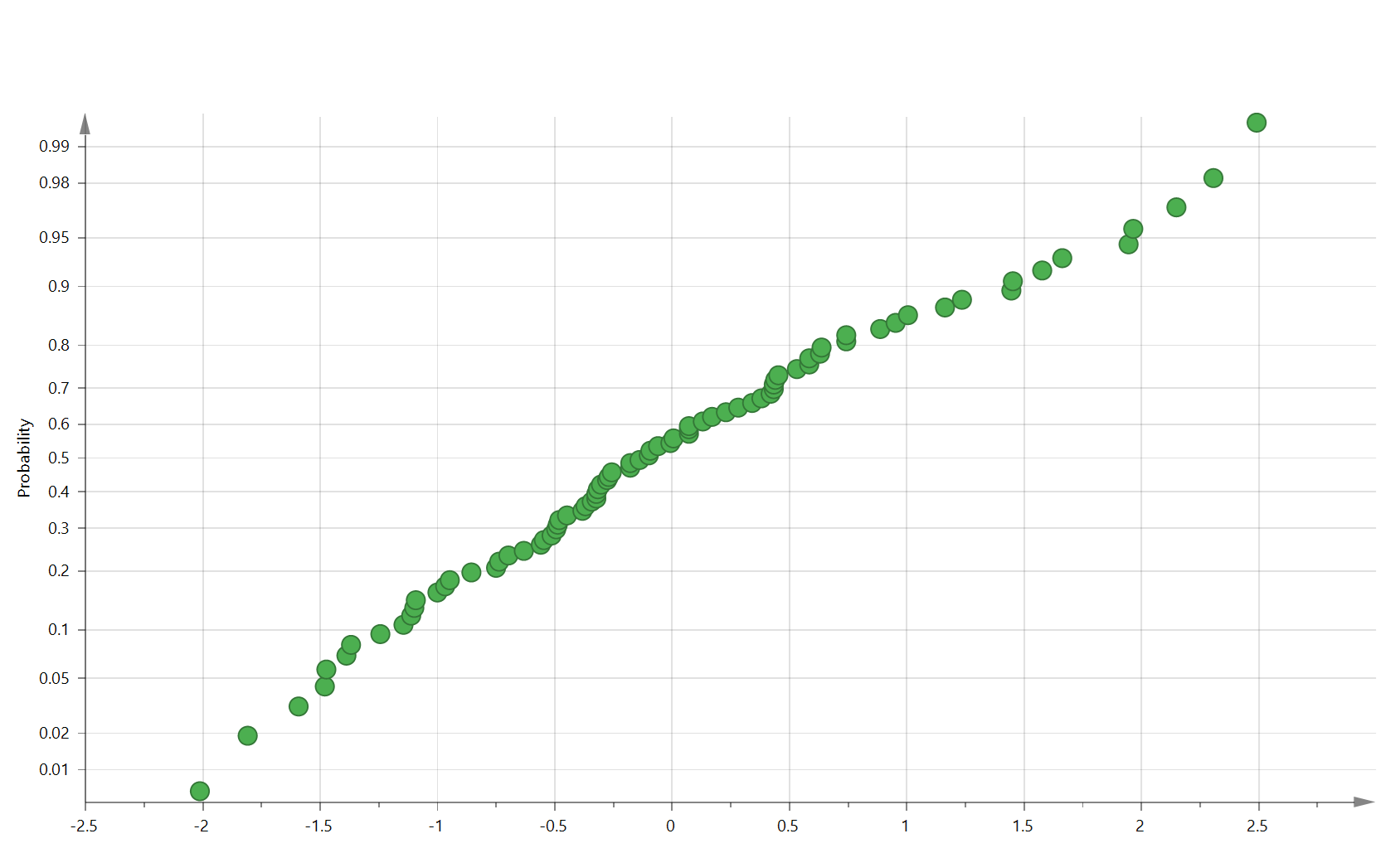


**Figure S1.** Residual normal probability plot.


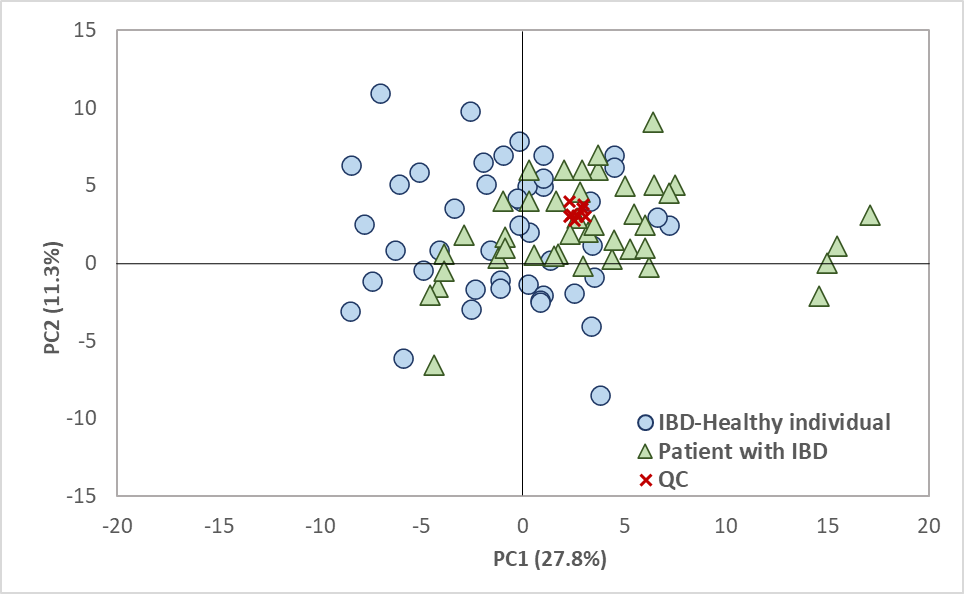


**Figure S2.** Score plot of PCA model (6 PCs) to differentiate between IBD-healthy individual and patient with IBD.


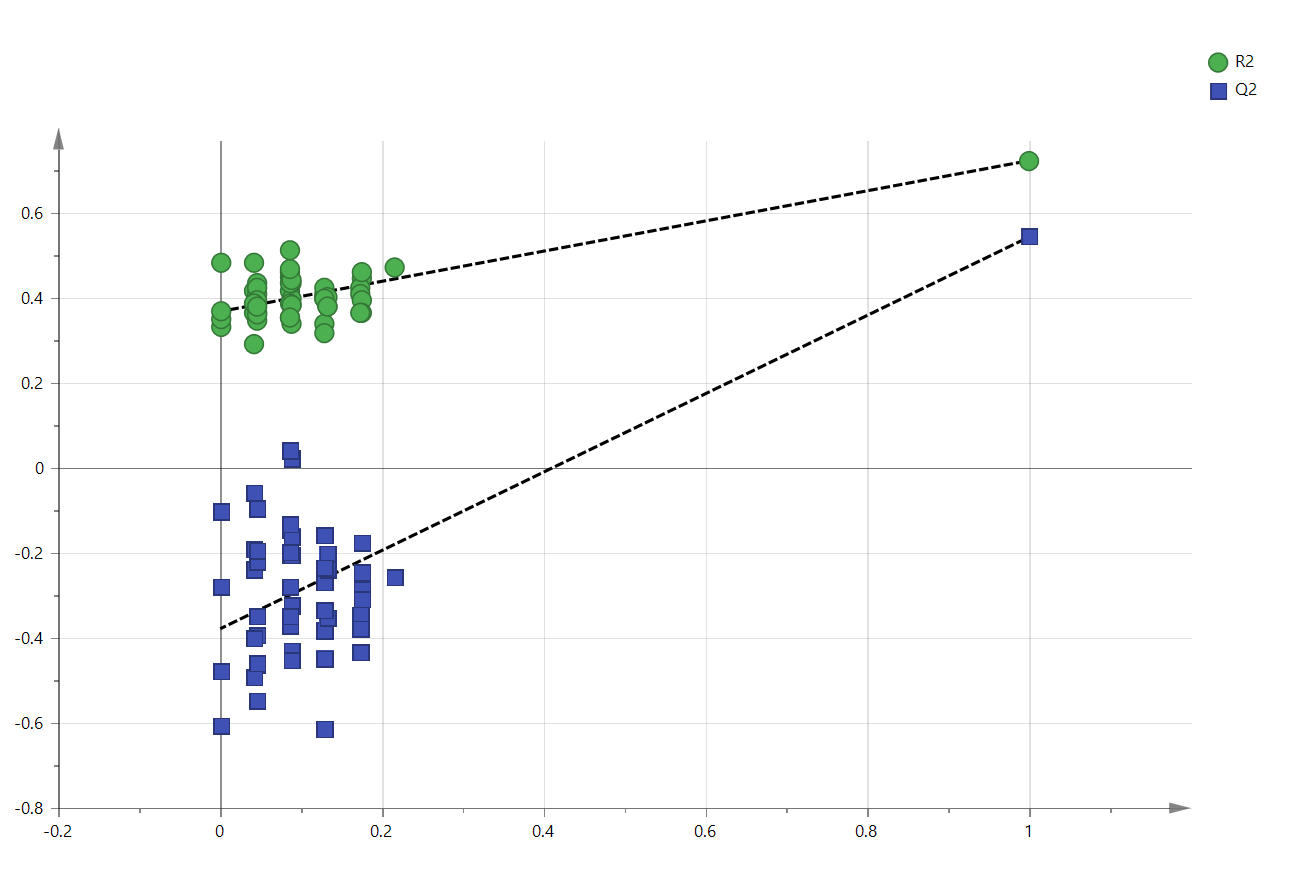


**Figure S3.** Permutations plot for OPLS-DA model using 50 random permutations


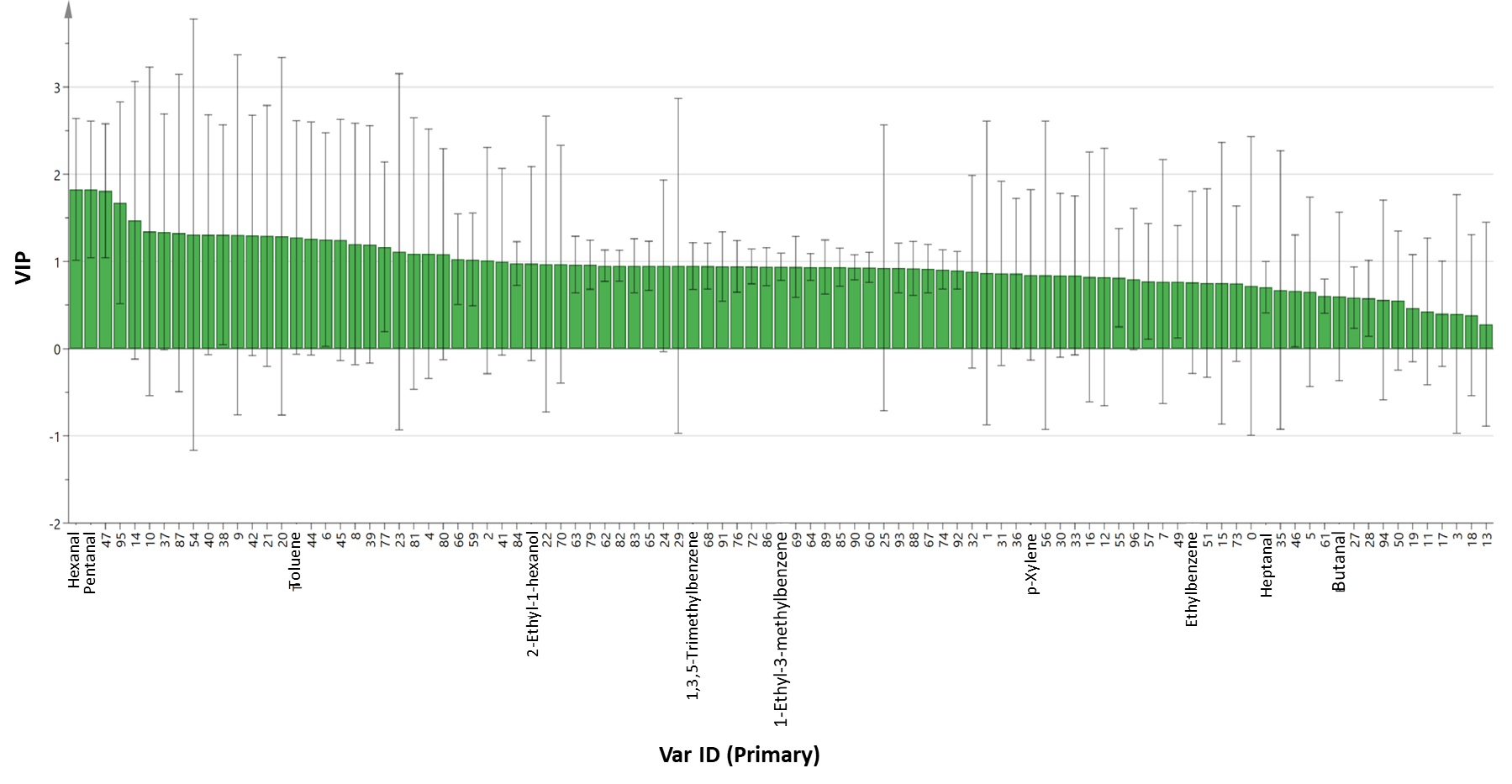


**Figure S4.** Variable Influence on the Projections (VIP) plot for IBD patient classification.


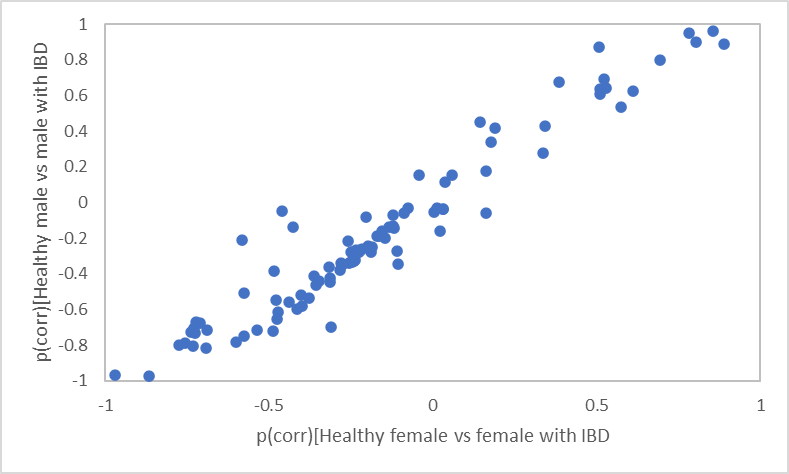


**Figure S5.** Shared and Unique Structures (SUS) plot analysis comparing OPLS gender - specific models for IBD-healthy versus patient with IBD for female (x-axis) and male (y-axis) gender.

**Table S1.** Results from CV-ANOVA’s of OPLS-DA model

|  | **SS** | **DF** | **MS** | **F** | **P** | **SD** |
| --- | --- | --- | --- | --- | --- | --- |
| **Total Corr.** | 80 | 80 | 1 |  |  | 1 |
| **Regression** | 49.4318 | 8 | 6.17898 | 14.5539 | 2.06089e-012 | 2.48575 |
| **Residual** | 30.5682 | 72 | 0.424558 |  |  | 0.651581 |

**Supplemental information of PLS-DA model**

2 components, R2X = 0.749, R2Y = 0.675 and Q2 = 0.522

**Table.** Results from CV-ANOVA’s of PLS-DA model

|  | **SS** | **DF** | **MS** | **F** | **P** | **SD** |
| --- | --- | --- | --- | --- | --- | --- |
| **Total Corr.** | 80 | 80 | 1 |  |  | 1 |
| **Regression** | 26.974 | 4 | 9.24349 | 16.3275 | 1.07897e-009 | 3.04031 |
| **Residual** | 43.026 | 76 | 0.566132 |  |  | 0.752417 |


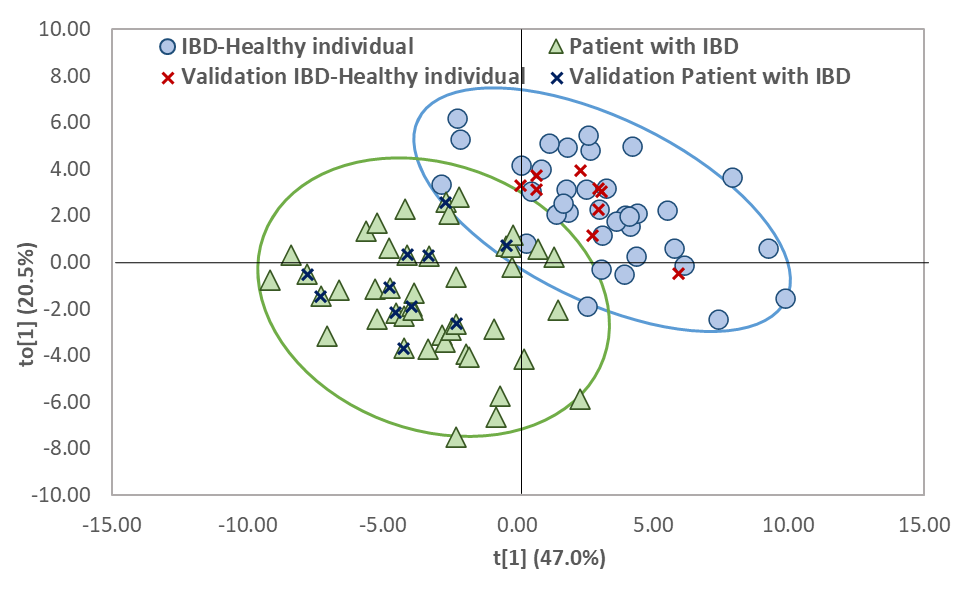


**Figure.** PLS-DA score plot for the classification of serum samples of IBD-healthy and IBD-patients including validation set (predicted samples). Ellipses for each category using a confidence probability level of 95% are shown.
